# Synthesis and Antimicrobial Evaluation of Some Novel Bis-α,β-Unsaturated Ketones, Nicotinonitrile, 1,2-Dihydropyridine-3-carbonitrile, Fused Thieno[2,3-*b*]pyridine and Pyrazolo[3,4-*b*]pyridine Derivatives

**DOI:** 10.3390/ijms14022967

**Published:** 2013-01-30

**Authors:** Farag M. A. Altalbawy

**Affiliations:** Department of Measurements and Environmental Applications, National Institute of Laser Enhanced Sciences (NILES), Cairo University, Giza 12613, Egypt; E-Mail: F_altalbawy@yahoo.com; Tel.: +20-100-562-7258; Fax: +202-570-8480

**Keywords:** heterocycles, α,β-unsaturated ketones, pyrazoles, antimicrobial activity

## Abstract

The title compounds were prepared by reaction of 1,1′-(5-methyl-1-phenyl-1*H-*pyrazole-3,4-diyl)diethanone (**1**) with different aromatic aldehydes **2a**–**c**, namely Furfural (**2a**), 4-chlorobenzaldehyde (**2b**) and 4-methoxybenzaldhyde (**2c**) to yield the corresponding α,β-unsaturated ketones **3a**–**c**. Compound **3** was reacted with malononitrile, 2-cyanoacetamide or 2-cyanothioacetamide yielded the corresponding bis[2-amino-6-(aryl)nicotinonitrile] **4a**–**c**, bis[6-(2-aryl)-2-oxo-1,2-dihydropyridine-3-carbonitrile] **5a**–**c** or bis[6-(2-aryl)-2-thioxo-1,2-dihydropyridine-3-carbonitrile] **6a**,**b**, respectively. The reaction of compound **6a** with each of 2-chloro-*N*-(4-bromophenyl) acetamide (**7a**), chloroacetamide (**7b**) in ethanolic sodium ethoxide solution at room temperature to give the corresponding 4,4′-(5-methyl-1-phenyl-1*H*-pyrazole-3,4-diyl)bis-6-(2-furyl)thieno[2,3-*b*]pyridine-2-carboxamide] derivatives **9a**,**b**. While compound **6a** reacted with hydrazine hydrate yielded the 4,4′-(5-methyl-1-phenyl-1*H*-pyrazole-3,4-diyl)bis[6-(2-furyl)-1*H*-pyrazolo[3,4-*b*]pyridin-3-amine] **11**. The structures of the products were elucidated based on their spectral properties, elemental analyses and, wherever possible, by alternate synthesis. Antimicrobial evaluation of the products was carried out.

## 1. Introduction

Recently, the synthesis of a wide variety of bis heterocyclic compounds of different ring sizes with one or several heteroatoms has received a great deal of attention, not only as model compounds for main chain polymers but also because many biologically active natural and synthetic products have molecular symmetry [1–8]. In addition, α,β-unsaturated ketones (Chalcones) derivatives have a variety of pharmacological activities such as antimalarial [9], anticancer [10–13], anti-inflammatory [14], antibacterial [15], antifungal agents [16]. Some nicotinonitrile derivatives are used as non-linear optical (NLO) materials [17], electrical materials [18]. Others have found uses as anticancer agents and Antimicrobial activity [19]. In addition, dihydropyridine derivatives display a broad spectrum of medicinal activities, mainly as antihypertensive and antiarrhythmic drugs [20–24]. Others have found uses as anticancer agents [25–27].

On the other hand, many compounds with pyrazole ring are of interest due to their broad spectrum of biological activities against NOS inhibitor [28], monoamine oxidase inhibitor [29], and antibacterial [30], antiamoebic [31]. Moreover, *N*-phenylpyrazole derivatives play an important role in antitumor screening [32] as well as potent antimicrobial activity [33,34]. Furthermore, a pyrazolo[3,4-*b*]pyridines have Potential and selective inhibitors of glycogen synthase kinase-3 (GSK-3) [35]. Also 3-Cyano-2(1*H*)-pyridinethiones [36,37] and their related compounds were found to be very reactive substances for the synthesis of many different heterocyclic systems which exhibited biological activities such as antibacterial and antifungal [38]. In light of these findings, we report here the synthesis of some novel bis-heterocycles containing *N*-phenylpyrazole as a base unit. In addition, some of the newly synthesized compounds were screened for their antibacterial and antifungal activities.

## 2. Results and Discussion

**Scheme 1:**
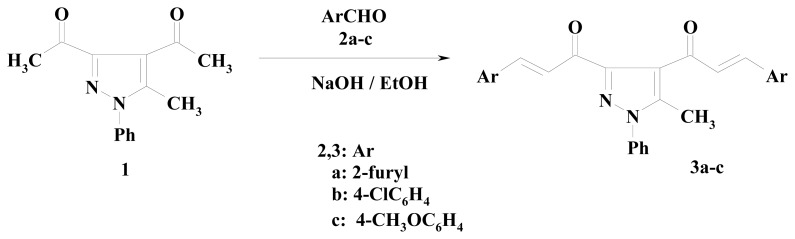
Synthesis of α,β-unsaturated ketones **3a**–**c**.

α,β-Unsaturated ketons (chalcones) are active intermediates and excellent starting materials for the synthesis of several heterocyclic systems. Thus, a Claisen-Schimdt reaction of 1,1′-(5-methyl-1-phenyl-1*H*-pyrazole-3,4-diyl)diethanone (**1**), prepared following the previously reported methods [39–41], with Furfural (**2a**), 4-chlorobenzaldehyde (**2b**) and 4-methoxybenzaldhyde (**2c**), in 10% ethanolic sodium hydroxide afforded the corresponding bis(α,β-unsaturated ketons) **3a**–**c** in 65, 95 and 50% yields, respectively (Scheme 1) [42].

The structure of the products **3a**–**c** was determined from spectroscopic as well as elemental analytical data. The IR spectra of **3a**–**c** showed the appearance of carbonyl function absorption bands in the region 1663–1658 cm^−1^. The ^1^H-NMR spectra of **3a**–**c** displayed the two signals of olefinic protons besides the aromatic protons [42], Thus, compound **3b**, taken as a typical example of the prepared series, revealed absorption bands at 2923, 1663 and 1598 cm^−1^ corresponding to CH-aliphatic, two carbonyl groups and C=C functions, respectively. Its ^1^H-NMR spectrum showed signals at δ 2.38 due to CH_3_ protons, four duplet signals at δ 7.24 (*J* = 11.3 Hz), 7.38 (*J* = 10.5 Hz), 7.54 (*J* = 10.5 Hz), 7.62 (*J* = 11.3 Hz) due to 4 CH = protons, in addition to an aromatic multiplet protons in the region δ 7.66–7.84. Their mass spectra revealed in each case the respective molecular ion peak.

**Scheme 2:**
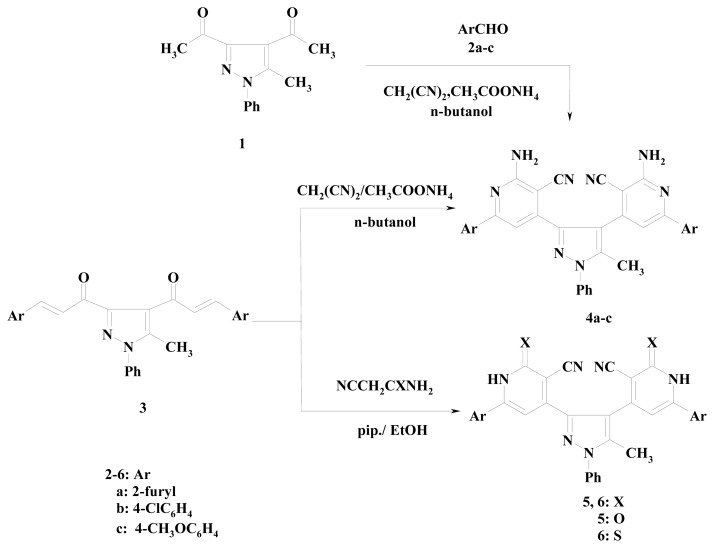
Synthesis of bis[2-amino-6-(aryl)nicotinonitrile] **4a**–**c**, bis[6-(2-aryl)-2-oxo-1,2-dihydropyridine-3-carbonitrile] **5a**–**c** and bis[6-(2-aryl)-2-thioxo-1,2-dihydropyridine-3-carbonitrile] **6a**,**b**.

Next, condensation of compounds **3a**–**c** with malononitrile in the presence of ammonium acetate in *n*-butanol afforded the corresponding 4,4′-(5-methyl-1-phenyl-1*H*-pyrazole-3,4-diyl) bis[2-amino-6-(aryl)nicotinonitrile] **4a**–**c** were also prepared in fair yields by applying the aforementioned one pot reaction of the compound **1**, the corresponding aldehyde and malononitrile in the presence of ammonium acetate (Scheme 2).

The structures of the newly synthesized compounds were confirmed on the basis of their elemental analysis and IR, ^1^HNMR and mass spectral data. The IR spectrum of compound **4b**, taken as a typical example of the series prepared, reveled absorption bands at 2210 and 3438, 3350 cm^−1^ corresponding to nitrile and NH_2_ function, respectively. Its ^1^HNMR spectrum showed two singlet signals at δ 2.32 and 7.35 due to CH_3_ and 2NH_2_ protons in addition to an aromatic multiplet in the region δ 7.38–7.61. Their mass spectra revealed in each case the respective molecular ion peak.

**Scheme 3:**
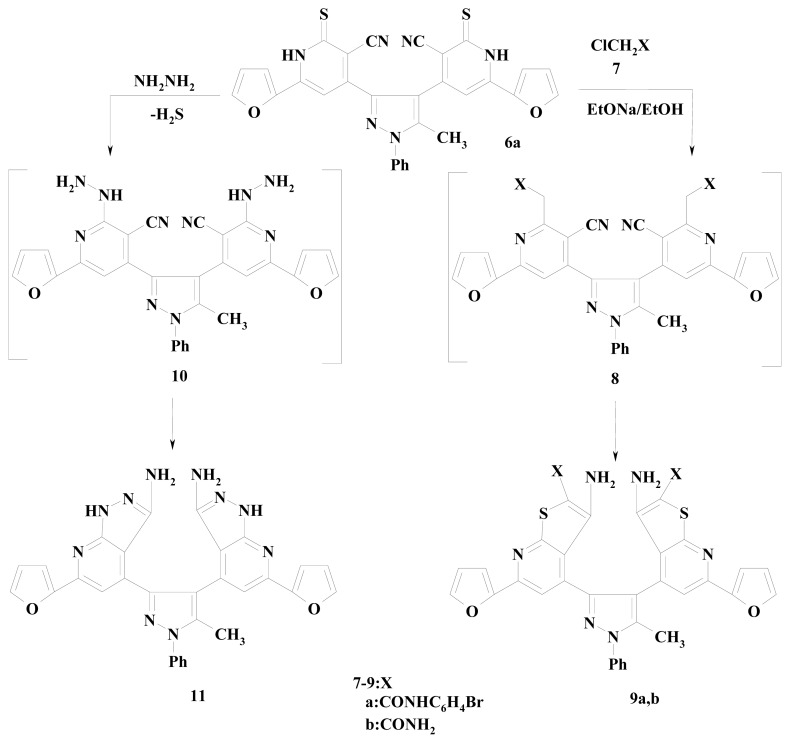
4,4′-(5-methyl-1-phenyl-1*H*-pyrazole-3,4-diyl)bis-6-(2-furyl)thieno[2,3-*b*]pyridine-2-carboxamide] derivatives **9a**,**b** and 4,4′-(5-methyl-1-phenyl-1*H*-pyrazole-3,4-diyl)bis[6-(2-furyl)-1*H*-pyrazolo[3,4-*b*]pyridin-3-amine] **11**.

But, **3a**–**b** was condensed with 2-cyanoacetamide and 2-cyanothioacetamide in the presence of pipridine in ethanol to yield the bis pyridine carbonitrile derivatives **5a**–**c** in 60%, 35% and 70% yields, respectively and bis pyridnethione derivatives **6a**,**b** in 60% and 55% yields, respectively, as shown in Scheme 2. The structures of the newly synthesized compounds were confirmed based on their elemental analysis, IR, ^1^HNMR and mass spectral data (see experimental). Compound **6a** was taken as an example reacted with each of 2-chloro-*N*-(4-bromophenyl) acetamide (**7a**), chloroacetamide (**7b**) by stirring in ethanolic sodium ethoxide solution at room temperature to give the corresponding 4,4′-(5-methyl-1-phenyl-1*H*-pyrazole-3,4-diyl)bis-6-(2-furyl)thieno[2,3-*b*]pyridine-2-carboxamide derivatives **9a**,**b**. However, treatment of the compound **6a** with hydrazine hydrate by refluxing in dioxane afforded the 4,4′-(5-methyl-1-phenyl-1*H*-pyrazole-3,4-diyl)bis[6-(2-furyl)-1*H-*pyrazolo[3,4-*b*]pyridin-3-amine] (**11**) as shown in Scheme 3. The structure of the products **9a**,**b** and **11** was elucidated by considering the data of IR, ^1^HNMR, mass spectra and elemental analyses.

## 3. Experimental Section

### 3.1. General Experimental Procedures

All melting points were measured on an Electrothermal Gallenkamp apparatus (Weiss-Gallenkamp, London, UK). The infrared spectra were recorded in potassium bromide discs on a Pye Unicam SP3300 and Shimadzo FT IR 8101 PC infrared spectrophotometers (Pye Unicam Ltd. Cambridge, England and Shimadzu, Tokyo, Japan, respectively). The ^1^H-NMR spectra were recorded on a Varian Mercury VXR-300 spectrometer (300 MHz). The mass spectra were recorded on a GCMS-Q1000-EX Shimadzu and GCMS 5988-A HP spectrometers, the ionizing voltage was 70 eV. Elemental analyses were carried out at the Micro-analytical Center of Cairo University, Giza, Egypt. The biological evaluation of the products was carried out in the Microbiology Division of Micro-analytical Center of Cairo University. The starting material Pyrazole **1** was prepared as previously reported in the literature [39,41].

### 3.2. Synthetic Procedures

#### 3.2.1. 1,1′-(5-Methyl-1-phenyl-1*H*-pyrazole-3,4-diyl)bis[3-Aryl prop-2-en-1-one] (**3a**–**c**)

A mixture of 1,1′-(5-methyl-1-phenyl-1*H*-pyrazole-3,4-diyl)diethanone (**1**) (4.84 g, 20 mmol), the appropriate aldehyde **2a**–**c** (40 mmol) and 10% ethanolic sodium hydroxide solution (15 mL) in ethanol (30 mL) was stirred for 12 h. The reaction mixture was then warmed at 40 °C for 10 min. and the separated precipitate was filtered off and recrystallized from ethanol to afford the corresponding compounds **3a**–**c**.

#### 3.2.2. 1,1′-(5-Methyl-1-phenyl-1*H*-pyrazole-3,4-diyl)bis[3-(2-furyl)prop-2-en-1-one] (**3a**)

Yield (65%), mp 180 °C (from EtOH); IR (KBr) ν_max_: 2916, 2852 (aliphatic CH), 1658 (C=O), 1595 (C=C) cm^−1; 1^H NMR (DMSO-d_6_): δ 2.35 (s, 3H, CH_3_), 6.60 (d, 1H, CH=, *J* = 10.06 Hz), 6.64 (d, 1H, CH=, *J* = 6.4 Hz), 7.05 (d, 1H, CH=, *J* = 10.06 Hz), 7.31 (d, 1H, CH=, *J* = 9.4 Hz), 7.26–7.89 (m, 11H, ArH). MS *m*/*z* (%): 398 (M^+^, 32.11), 277 (48.19), 265 (66.94), 230 (30.71), 213 (20.16), 154 (21.47). Anal. Calcd for C_24_H_18_N_2_O_4_ (398.41): C, 72.35; H, 4.55; N, 7.03. Found: C, 72.30; H, 4.52; N, 7.00%.

#### 3.2.3. 1,1′-(5-Methyl-1-phenyl-1*H*-pyrazole-3,4-diyl)bis[3-(4-chlorophenyl) prop-2-en-1-one] (**3b**)

Yield (95%), mp 230 °C (from Dioxane); IR (KBr) ν_max_: 3061 (aromatic CH), 2923 (aliphatic CH), 1663 (C=O), 1598 (C=C) cm^−1; 1^H NMR (DMSO-d_6_): δ 2.38 (s, 3H, CH_3_), 7.24 (d, 1H, CH=, *J* = 11.3 Hz), 7.38 (d, 1H, CH=, *J* = 10.5 Hz), 7.54 (d, 1H, CH=, *J* = 10.5 Hz), 7.62 (d, 1H, CH=, *J* = 11.3 Hz), 7.66–7.84 (m, 13H, ArH). MS *m*/*z* (%): 490 (M^+^ + 3, 4.86), 489 (M^+^ + 2, 7.68), 488 (M^+^ + 1, 23.38), 487 (M^+^, 14.66), 375 (7.36), 394 (11.66), 264 (0.96), 221 (0.33), 156 (2.20). Anal. Calcd for C_28_H_20_Cl_2_N_2_O_2_ (487.37): C, 69.00; H, 4.14; N, 5.75; Cl, 14.55%. Found: C, 68.97; H, 4.12; N, 5.72; Cl, 14.53%.

#### 3.2.4. 1,1′-(5-Methyl-1-phenyl-1*H*-pyrazole-3,4-diyl)bis[3-(4-methoxyphenyl)prop-2-en-1-one] (**3c**)

Yield (50%), mp 160 °C (from ethanol); IR (KBr) ν_max_: 3068 (aromatic CH), 2926, 2835 (aliphatic CH), 1661 (C=O), 1594 (C=C) cm^−1; 1^H NMR (DMSO-d_6_): δ 2.22 (s, 3H, CH_3_), 3.81 (s, 6H, 2OCH_3_), 6.96 (d, 1H, CH=, *J* = 9.8 Hz), 7.26 (d, 1H, CH=, *J* = 10.7 Hz), 7.40 (d, 1H, CH=, *J* = 10.7 Hz), 7.50 (d, 1H, CH=, *J* = 9.8 Hz), 7.52–7.79 (m, 13H, ArH). MS *m*/*z* (%): 479 (M^+^ + 1, 1.4), 478 (M^+^, 6.70), 371 (6.2), 345 (3.3), 317 (8.5), 264 (2.9), 212 (2.4), 156 (2.6). Anal. Calcd for C_30_H_26_N_2_O_4_ (478.53): C, 75.30; H, 5.48; N, 5.85%. Found: C, 75.28; H, 5.45; N, 5.84%.

#### 3.2.5. 4,4′-(5-Methyl-1-phenyl-1*H*-pyrazole-3,4-diyl)bis[2-amino-6-(aryl)nicotinonitrile] (**4a**–**c**)

Method A: A mixture of 1,1′-(5-methyl-1-phenyl-1*H*-pyrazole-3,4-diyl)diethanone (**1**) (4.84 g, 20 mmol), malononitrile (2.64 g, 40 mmol), the appropriate aldehyde **2a**–**c** (40 mmol) and ammonium acetate (6.0 g), was heated under reflux in *n*-butanol (40 mL) for 3 h. On cooling, the separated yellow solid was filtered, washed with water and crystallized.

Method B: A mixture of each of 1,1′-(5-methyl-1-phenyl-1*H*-pyrazole-3,4-diyl)bis[3-Aryl prop-2-en-1-one] **3a**–**c** (1 mmol), malononitrile (2 mmol), and 0.616 g ammonium acetate (8 mmol) in *n*-butanol (40 mL) was refluxed for 3 h. After cooling, the precipitate was filtered off, dried, and crystallized to give **4a**–**c**.

#### 3.2.6. 4,4′-(5-Methyl-1-phenyl-1*H*-pyrazole-3,4-diyl)bis[2-amino-6-(2-furyl)nicotinonitrile] (**4a**)

Yield (50%), mp 230 °C (from ethanol); IR (KBr) ν_max_: 3438, 3376 (NH_2_), 2203 (C≡N) cm^−1; 1^H NMR (DMSO-d_6_): δ 2.39 (s, 3H, CH_3_), 6.72 (s, 4H, 2NH_2_), 7.11–7.96 (m, 13H, ArH). MS *m*/*z* (%): 526 (M^+^ + 2, 3.87), 525 (M^+^ + 1, 2.4214), 524 (M^+^, 100), 468 (45), 367 (7.51), 347 (2.48), 152 (3.05). Anal. Calcd for C_30_H_20_N_8_O_2_ (524.53): C, 68.69; H, 3.84; N, 21.36. Found: C, 68.67; H, 3.81; N, 21.35%.

#### 3.2.7. 4,4′-(5-Methyl-1-phenyl-1*H*-pyrazole-3,4-diyl)bis[2-amino-6-(4-chlorophenyl)nicotinonitrile] (**4b**)

Yield (40%), mp 210 °C (from ethanol); IR (KBr) ν_max_: 3438, 3350 (NH_2_), 2210 (C≡N) cm^−1; 1^H NMR (DMSO-d_6_) δ: 2.32 (s, 3H, CH_3_), 7.35 (s, 4H, 2NH_2_), 7.38–7.61 (m, 15H, ArH). MS *m*/*z* (%): 615 (M^+^ + 2, 6.6), 614 (M^+^ + 1, 6.6), 613 (M^+^, 0.8), 456 (5.3), 384 (5.3), 153 (23.7), 118 (25). Anal. Calcd for C_34_H_22_Cl_2_N_8_ (613.49): C, 66.56; H, 3.61; N, 18.26; Cl, 11.56. Found: C, 66.54; H, 3.60; N, 18.25; Cl, 11.52%.

#### 3.2.8. 4,4′-(5-Methyl-1-phenyl-1*H*-pyrazole-3,4-diyl)bis[2-amino-6-(4-methoxyphenyl)nicotinonitrile] (**4c**)

Yield (45%); mp 200 °C (from ethanol); IR (KBr) ν_max_: 3456–3347 (NH_2_), 2200 (C≡N) cm^−1; 1^H-NMR (DMSO-d_6_) (ppm): 2.47 (s, 3H, CH_3_), 3.83 (s, 6H, 2OCH_3_), 7.06 (s, 4H, 2NH_2_), 7.33–7.64 (m, 15H, ArH’s); ^13^C-NMR: δ 30.4, 55.3, 84.6, 88.4, 104.2, 108.6, 109.6, 113.9, 114.3, 117.5, 121.7, 125.2, 126.7, 127.2, 128.2, 129.4, 129.9, 130.5, 133.2, 135.6, 139.2, 145.6, 146.5, 153.3, 155.6, 160.4, 160.9, 163.8; MS *m*/*z* (%): 604 (M^+^, 14), 347 (33.3), 257 (37), 119 (22.2). Anal. Calcd for C_36_H_28_N_8_O_2_ (604.68): C, 71.51; H, 4.67; N, 18.53. Found: C, 71.50; H, 4.65; N, 18.50%.

#### 3.2.9. 4,4′-(5-Methyl-1-phenyl-1*H*-pyrazole-3,4-diyl)bis[6-(2-aryl)-2-oxo-1,2-dihydropyridine-3-carbonitrile] (**5a**–**c**)

Method A: A mixture of each of 1,1′-(5-methyl-1-phenyl-1*H*-pyrazole-3,4-diyl)bis[3-aryl prop-2-en-1-one] **3a**–**c** (1 mmol), cyanoacetamide (0.17 g, 2 mmol), and (1.21 g, 16 mmol) ammonium acetate in *n*-butanol (40 mL) was refluxed for 3 h. After cooling, the precipitate was filtered off, dried, and crystallized to give **5a**–**c**.

Method B: A mixture of 1,1′-(5-methyl-1-phenyl-1*H*-pyrazole-3,4-diyl)diethanone (**1**) (0.24 g, 1 mmol), appropriate aldehyde (2 mmol), cyanoacetamide (0.17 g, 2 mmol) and (1.21 g, 16 mmol) ammonium acetate in *n*-butanol (40 mL) was refluxed for 4 h. After cooling, the formed product was collected by filtration and crystallized to give **5a**–**c**.

#### 3.2.10. 4,4′-(5-Methyl-1-phenyl-1*H*-pyrazole-3,4-diyl)bis[6-(2-furyl)-2-oxo-1,2-dihydropyridine-3-carbonitrile] (**5a**)

Yield (60%); mp 220 °C (from ethanol); IR (KBr) ν_max_: 3355 (NH), 2213 (C≡N), 1655 (C=O) cm^−1; 1^H NMR (DMSO-d_6_) (ppm): 2.35 (s, 3H, CH_3_), 8.40 (s, 2H, 2NH), 6.91–7.51 (m, 13H, ArH); ^13^C-NMR: δ 22.1, 90.4, 92.5, 95.0, 104.8, 110.4, 112.3, 114.1, 114.7, 115.6, 116.9, 123.1, 124.5, 129.5, 130.4, 137.8, 140.9, 142.0, 143.2, 143.9, 145.1, 146.7, 149.22, 152.3, 152.61, 153.2, 156.8; MS *m/z* (%): 527 (M^+^ + 1, 1.19), 526 (M^+^, 2.26), 458 (0.02), 391 (3.08), 370 (16.12), 156 (7.36), 118 (41.54), 67 (13.06); Anal. Calcd for C_30_H_18_N_6_O_4_ (526.52): C, 68.44; H, 3.45; N, 15.96. Found: C, 68.40; H, 3.41; N, 15.95.

#### 3.2.11. 4,4′-(5-Methyl-1-phenyl-1*H*-pyrazole-3,4-diyl)bis[6-(4-chlorophenyl)-2-oxo-1,2-dihydropyridine-3-carbonitrile] (**5b**)

Yield (35%), mp 202 °C (from ethanol); IR (KBr) ν_max_: 3436 (NH), 2216 (C≡N), 1652 (C=O); ^1^H NMR (DMSO-d_6_): δ 2.37 (s, 3H, CH_3_), 7.71 (s, 2H, 2NH), 7.25–7.51 (m, 15H, ArH). MS *m*/*z* (%): 615 (M^+^, 5.40), 385 (10.9), 154 (8.7), 119 (15.2). Anal. Calcd for C_34_H_20_Cl_2_N_6_O_2_ (615.48): C, 66.35; H, 3.28; N, 13.65; Cl, 11.52. Found: C, 66.32; H, 3.25; N, 13.60; Cl, 11.50%.

#### 3.2.12. 4,4′-(5-Methyl-1-phenyl-1*H*-pyrazole-3,4-diyl)bis[6-(4-methoxyphenyl)-2-oxo-1,2-dihydropyridine-3-carbonitrile] (**5c**)

Yield (70%), mp 190 °C (from ethanol); IR (KBr) ν_max_: 3437 (NH), 2216 (C≡N), 1664 (C=O) cm^−1; 1^H NMR (DMSO-d_6_): δ 2.36 (s, 3H, CH_3_), 3.83 (s, 6H, 2OCH_3_), 7.65 (s, 2H, 2NH), 7.10–7.61 (m, 15H, ArH). MS m/z (%): 606 (M^+^, 1.9), 224 (3.7), 156 (3.7), 119 (18.1), 106 (4.2). Anal. Calcd for C_36_H_26_N_6_O_4_ (606.65): C, 71.28; H, 4.32; N, 13.85. Found: C, 71.25; H, 4.30; N, 13.82%.

#### 3.2.13. 4,4′-(5-Methyl-1-phenyl-1*H*-pyrazole-3,4-diyl)bis[6-(2-furyl)-2-thioxo-1,2-dihydropyridine-3-carbonitrile] (**6a**,**b)**

An equimolecular amount of 1,1′-(5-methyl-1-phenyl-1*H*-pyrazole-3,4-diyl)bis[3-(2-furyl)prop-2-en-1-one] (**3a**) (0.334 g, 1 mmol), 2-cyanoethanethioamide (0.2 g, 2 mmol) and few drops of piperidine in Ethanol (30 mL) was heated under reflux for 5 h. After cooling, the solid product was collected by filtration, washed with ethanol and then recrystallized from ethanol to give **6a**,**b**.

#### 3.2.14. 4,4′-(5-Methyl-1-phenyl-1*H*-pyrazole-3,4-diyl)bis[6-(2-furyl)-2-thioxo-1,2-dihydropyridine-3-carbonitrile] (**6a**)

Yield (60%), mp 260 °C (from ethanol); IR (KBr) ν_max_: 3425 (NH), 3053 (aromatic CH), 2924, 2853 (aliphatic CH), 2216 (C≡N) cm^−1; 1^H NMR (DMSO-d_6_): δ 2.37 (s, 3H, CH_3_), 7.98 (s, 2H, 2NH), 6.67–7.82 (m, 13H, ArH). MS *m*/*z* (%): 558 (M^+^, 1.2), 374 (81.8), 287 (22.7), 227 (50), 127 (22.7), 91 (59.1), 67 (22.7). Anal. Calcd for C_30_H_18_N_6_O_2_S_2_ (558.64): C, 64.50; H, 3.25; N, 15.04; S, 11.48. Found: C, 64.48; H, 3.24; N, 15.00; S, 11.46%.

#### 3.2.15. 4,4′-(5-Methyl-1-phenyl-1*H*-pyrazole-3,4-diyl)bis[6-(4-chlorophenyl)-2-thioxo-1,2-dihydropyridine-3-carbonitrile] (**6b**)

Yield (55%); mp 250 °C (from ethanol); IR (KBr) ν_max_: 3416 (NH), 3048 (aromatic CH), 2847 (aliphatic CH), 2206 (C≡N); ^1^H NMR (DMSO-d_6_) (ppm): δ 2.40 (s, 3H, CH_3_), 8.00 (s, 2H, 2NH), 7.46–7.85 (m, 15H, ArH); ^13^C-NMR: δ 21.5, 43.7, 94.3, 98.4, 102.3, 115.2, 120.8, 121.7, 124.1, 125.0, 128.2, 129.4, 130.1, 130.8, 131.6, 132.4, 133.5, 136.7, 139.7, 140.1, 142.6, 151.86, 157.5, 160.2, 161.8, 184.3, 188.0; MS m/z (%): 649 (M^+^ + 2, 1.8), 648 (M^+^ + 1, 2.2), 647 (M^+^, 3.5), 528 (11.6), 354 (11.6), 313 (23.3), 114 (20.9), 64 (100); Anal. Calcd for C_34_H_20_Cl_2_N_6_S_2_ (647.61): C, 63.06; H, 3.11; N, 12.98; Cl, 10.95; S, 9.90. Found: C, 63.02; H, 3.10; N, 12.95; Cl, 10.93; S, 9.89%.

#### 3.2.16. Synthesis of Compounds **9a**,**b**

*General Procedure*: A solution of each of **6a** (0.558 g, 1 mmol and 2-chloro-*N*-(4-bromophenyl)-acetamide (**7a**) (0.497 g, 2 mmol) or 2-chloroacetamide (**7b**) (0.187 g, 2 mmol) in sodium methoxide (prepared from 0.10 g of sodium and ethanol 25 mL) was stirred at room temperature for 15 min. The formed precipitate was collected by filtration, washed with water, ethanol and dried, to give **9a**,**b** respectively.

#### 3.2.17. 4,4′-(5-Methyl-1-phenyl-1*H*-pyrazole-3,4-diyl)bis[3-amino-*N*-(4-bromophenyl)-6-(2-furyl) thieno[2,3-*b*]pyridine-2-carboxamide] (**9a**)

Yield (50%); mp 180 °C (from ethanol); IR (KBr) ν_max_: 3475–3328, 3111 (NH and NH_2_), 1641 (C=O) cm^−1; 1^H-NMR (DMSO-d_6_) (ppm): δ 2.34 (s, 3H, CH_3_), 6.96 (s, 4H, 2NH_2_), 7.21–8.06 (m, 21H, ArH), 9.55 (s, 2H, 2NH); ^13^C-NMR: δ 23.7, 48.3, 105.1, 107.1, 109.2, 110.1, 111.2, 112.3, 112.5, 115.2, 120.9, 122.9, 125.2, 126.2, 127.3, 128.5, 129.3, 130.2, 131.1, 133.8, 137.3, 137.8, 138.2, 138.9, 139.6, 145.4, 147.4, 155.6, 157.4, 158.0, 159.0, 160.3, 161.5, 163.7, 165.2, 165.9; MS *m*/*z* (%): 984 (M^+^ + 2, 33), 983 (M^+^ + 1, 50), 982 (M^+^, 25), 953 (41), 783 (50), 588 (58), 156 (58); Anal. Calcd for C_46_H_30_Br_2_N_8_O_4_S_2_ (982.72): C, 56.22; H, 3.08; N, 11.40; S, 6.53; Br, 16.26. Found: C, 56.20; H, 3.00; N, 11.38; S, 6.50; Br, 16.23.

#### 3.2.18. 4,4′-(5-Methyl-1-phenyl-1*H*-pyrazole-3,4-diyl)bis[3-amino-6-(2-furyl) thieno[2,3-*b*]pyridine-2-carboxamide] (**9b**)

Yield (30%), mp 194 °C (from ethanol); IR (KBr) ν_max_: 3434, 3115 (NH and NH_2_), 3062 (aromatic CH), 2953 (aliphatic CH), 1681 (C=O) cm^−1; 1^H NMR (DMSO-d_6_): δ 2.40 (s, 3H, CH_3_), 6.62–6.68 (s, 4H, 2NH_2_), 7.13–7.87 (m, 17H, 2NH_2_ and 13H ArH). MS *m*/*z* (%): 672 (M^+^, 34). Anal. Calcd for C_34_H_24_N_8_O_4_S_2_ (672.75): C, 60.70; H, 3.60; N, 16.66; S, 9.53%. Found: C, 60.66; H, 3.59; N, 16.60; S, 9.50%.

#### 3.2.19. 4,4′-(5-Methyl-1-phenyl-1*H*-pyrazole-3,4-diyl)bis[6-(2-furyl)-1*H*-pyrazolo[3,4-*b*]pyridin-3-amine] (**11**)

A mixture of compound **6a** (5.58 g, 0.01 mol) and hydrazine hydrate (20–25 mL) was heated under reflux for 7 h. The solid product was filtered off, washed with EtOH, dried and crystallized from ethanol. Yield (35%), mp 265 °C (from ethanol); IR (KBr) ν_max_: 3314, 3194 (NH and NH_2_), 2967 (aliphatic CH) cm^−1; 1^H NMR (DMSO-d_6_): δ 2.49 (s, 3H, CH_3_), 5.01 (s, 4H, 2NH_2_), 6.66–7.99 (m, 13H, ArH), 8.66 (s, 2H, 2NH). MS m/z (%): 554 (M^+^, 0.2), 545 (9.4), 489 (26), 199 (15), 155 (15), 77 (100), 67 (11). Anal. Calcd for C_30_H_22_N_10_O_2_ (554.58): C, 64.97; H, 4.00; N, 25.26%. Found: C, 64.92; H, 3.98; N, 25.20%.

### 3.3. Antimicrobial Evaluation

The antibacterial and antifungal activity assays were carried out at the Microbiology Division of Microanalytical Center of Cairo University using the diffusion plate method [43–45]. A bottomless cylinder containing a measured quantity (1 mL, mg/mL) of the sample is placed on a plate (9 cm diameter) containing a solid bacterial medium (nutrient agar broth) or fungal medium, which has been heavily seeded with a spore suspension of the test organism. After incubation (24 h for bacteria and 5 days for fungi), the diameter of the clear zone of inhibition surrounding the sample is taken as measure of the inhibitory power of the sample against the particular test organism. The solvent used was DMSO and the concentration of the sample used is 100 μg/mL. The results of antimicrobial activity are summarized in Table 1. Most of the synthesized compounds were evaluated for their antibacterial against a Gram negative bacterium (*Escherichia coli* anaerobic (EC)), a Gram positive bacterium (*Staphylococcus aureus* (SA)) and for antifungal activity against *Candida albicans* (CA) and *Aspergillus flavus* (AF) by diffusion technique [43–45]. As seen from the data present in Table 1, *Escherichia coli* anaerobic is sensitive to compounds **3a**, **4c** and **9a**; furthermore, *Staphylococcus aureus* is sensitive to compounds **4b**,**c**, **5a** and **9a**. Whereas, all tested compounds did not exhibit the antifungal activity against the two tested fungi species *Candida albicans* and *Aspergillus flavus*. The activity of **4c** and **9a** is attributed to the presence of pharmacological active 4-methoxyphenyl at position 6 of the nicotinonitrile and 4-bromophenyl at position *N* of carboxamide.

## 4. Conclusions

In conclusion, the reactivity of 1,1′-(5-methyl-1-phenyl-1*H*-pyrazole-3,4-diyl)diethanone (**1**) was investigated as a versatile and readily accessible building block for the synthesis of new bis-heterocycles incorporating 5-methyl-1-phenyl-1*H*-pyrazole moiety of biological and pharmaceutical importance.

## Figures and Tables

**Table 1:** Antibacterial and antifungal activities of the synthesized compounds (**3a**–**c**, **4a**–**c**, **5a**,**c**, **6a**,**b**) and **9a**.

Compound No.	Inhibition Zone Diameter (cm)			

	Gram (−)	Gram (+)	Fungi	

	(EC)	(SA)	(AF)	(CA)
**3a**	10	0.0	0.0	0.0
**3b**	0.0	0.0	0.0	0.0
**3c**	0.0	0.0	0.0	0.0
**4a**	0.0	0.0	0.0	0.0
**4b**	0.0	12	0.0	0.0
**4c**	16	16	0.0	0.0
**5a**	0.0	10	0.0	0.0
**5c**	0.0	0.0	0.0	0.0
**6a**	0.0	0.0	0.0	0.0
**6b**	0.0	0.0	0.0	0.0
**9a**	13	14	0.0	0.0
Tetracycline	30	29		
Diflucan			18	21

ATCC for (EC, SA, SA and CA) are 11775, 12600 and 26555, respectively.
